# Supplementary material for: A “double-edged” role for type-5 metabotropic glutamate receptors in pain disclosed by light-sensitive drugs
Source: bioRxiv. 2024 Jan 3:2024.01.02.573945. Preprint. [Version 1] doi: 10.1101/2024.01.02.573945 (PMC10802266; doi:10.1101/2024.01.02.573945)
Supplement: 1 [file NIHPP2024.01.02.573945V1-supplement-1.pdf]

bioRxiv preprint doi: https://doi.org/10.1101/2024.01.02.573945; this version posted January 3, 2024. The copyright holder for this preprint (which was not certified by peer review) is the author/funder, who has granted bioRxiv a license to display the preprint in perpetuity. It is made available under aCC-BY 4.0 International license.

Supplementary Materials for

A "double edged" role for type-5 metabotropic glutamate receptors in pain disclosed by light-sensitive drugs

Serena Notartomaso *et al.*

*Corresponding author. Email: volker.neugebauer@ttuhsc.edu

This PDF file includes:

Figs. S1 to S3

bioRxiv preprint doi: https://doi.org/10.1101/2024.01.02.573945; this version posted January 3, 2024. The copyright holder for this preprint (which was not certified by peer review) is the author/funder, who has granted bioRxiv a license to display the preprint in perpetuity. It is made available under aCC-BY 4.0 International license.

Fig. S1

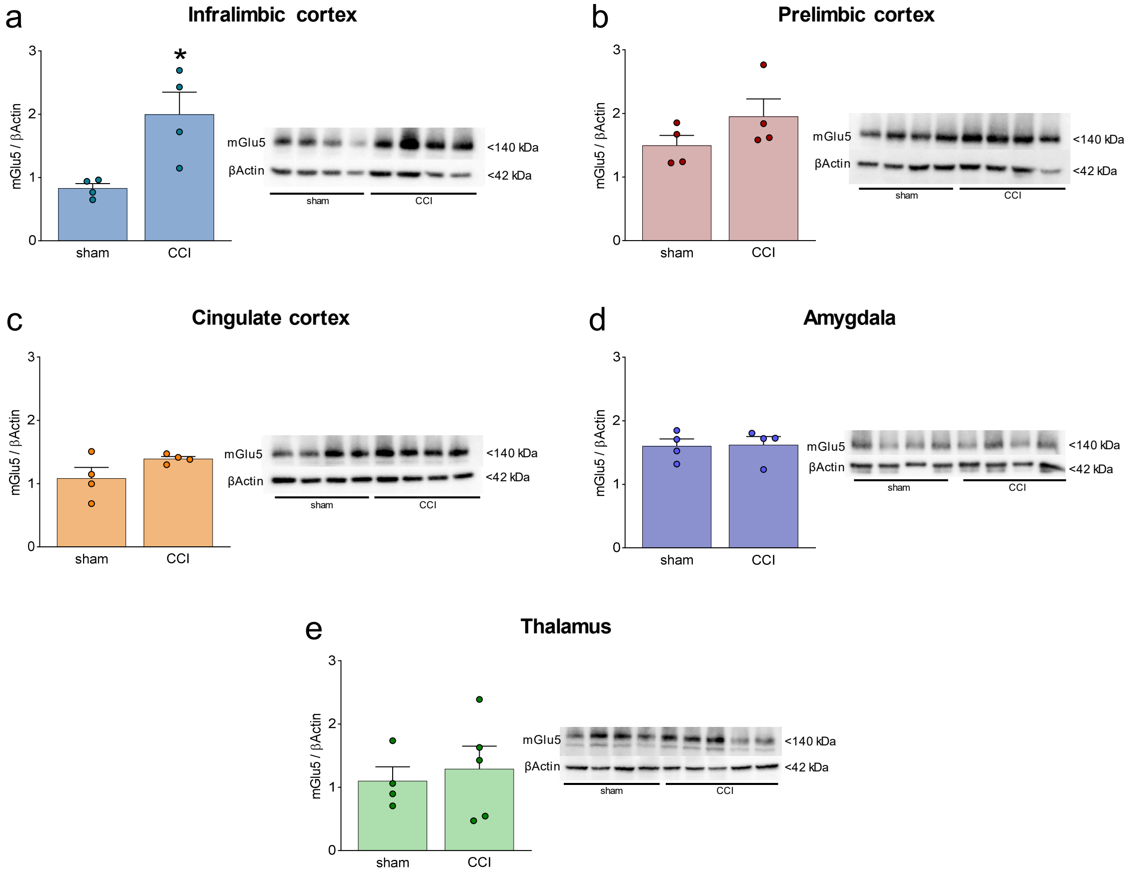

Fig. S1.

mGlu5 receptor protein levels in different brain regions in sham and CCI mice. (**a-e**) mGlu5 receptor protein levels in the contralateral (to the side of injury) infralimbic cortex (**a**), prelimbic cortex (**b**), anterior cingulate cortex (**c**), amygdala (**d**) and thalamus (**e**). Significant changes were found only in infralimbic cortex where CCI caused an increase in mGlu5 receptor protein levels compared to sham mice. Bar histograms show mean +SEM of 4-5 mice per group. *$p<0.05$, unpaired student's t-test compared to sham. (a) t=3265, p=0.0171; (b) t=1.425, p=0.02041; (c) t=1.764, p=0.01281; (d) t=0.1172, p=0.9105; (e) t=0.4257, p=0.06831.

bioRxiv preprint doi: https://doi.org/10.1101/2024.01.02.573945; this version posted January 3, 2024. The copyright holder for this preprint (which was not certified by peer review) is the author/funder, who has granted bioRxiv a license to display the preprint in perpetuity. It is made available under aCC-BY 4.0 International license.

Fig. S2

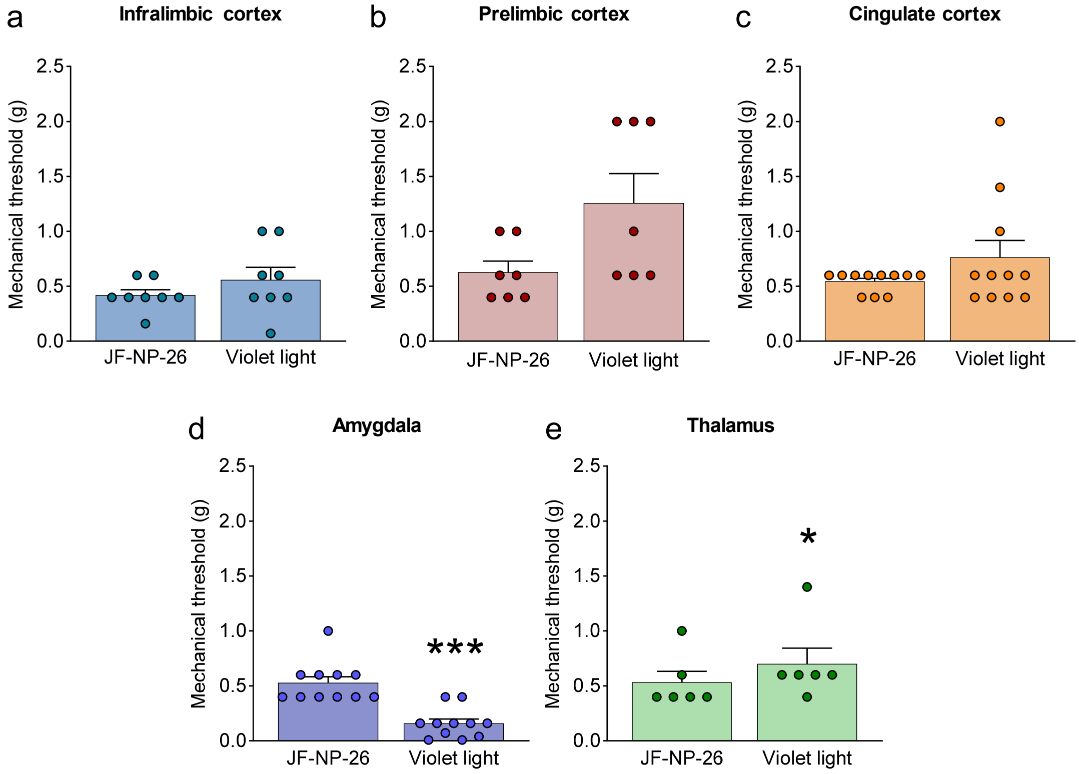

Fig. S2.

Effects of light-induced blockade of mGlu5 receptors in different brain regions on mechanical pain thresholds in the unlesioned paw of CCI mice. Mechanical thresholds were measured in the right paw (contralateral to the injury) of CCI mice before and after light-induced activation of systemic JF-NP-26 (10 mg/kg, i.p.) in the contralateral (to the side of injury) infralimbic cortex (**a**), prelimbic cortex (**b**), anterior cingulate cortex (**c**), amygdala (**d**) and thalamus (**e**). Blue-violet light activation of JF-NP-26 in prefrontal cortical regions had no effect on mechanical thresholds on the uninjured paw, but blue-violet light illumination in the thalamus was slightly antinociceptive and in the amygdala caused hypersensitivity. Bar histograms show mean + SEM of 8 (a), 7 (b), 11 (c), 11 (d) and 6 (e) mice per group. *$p<0.05$, ***$p<0.001$ unpaired student's t-test compared to JF-NP-26. (a) t=1.370, p=0.2129; (b) t=2.238, p=0.0666; (c) t=1.491, p=0.1669; (d) t=5.746, p=0.0002; (e) t=2.712, p=0.0422.

bioRxiv preprint doi: https://doi.org/10.1101/2024.01.02.573945; this version posted January 3, 2024. The copyright holder for this preprint (which was not certified by peer review) is the author/funder, who has granted bioRxiv a license to display the preprint in perpetuity. It is made available under aCC-BY 4.0 International license.

Fig. S3

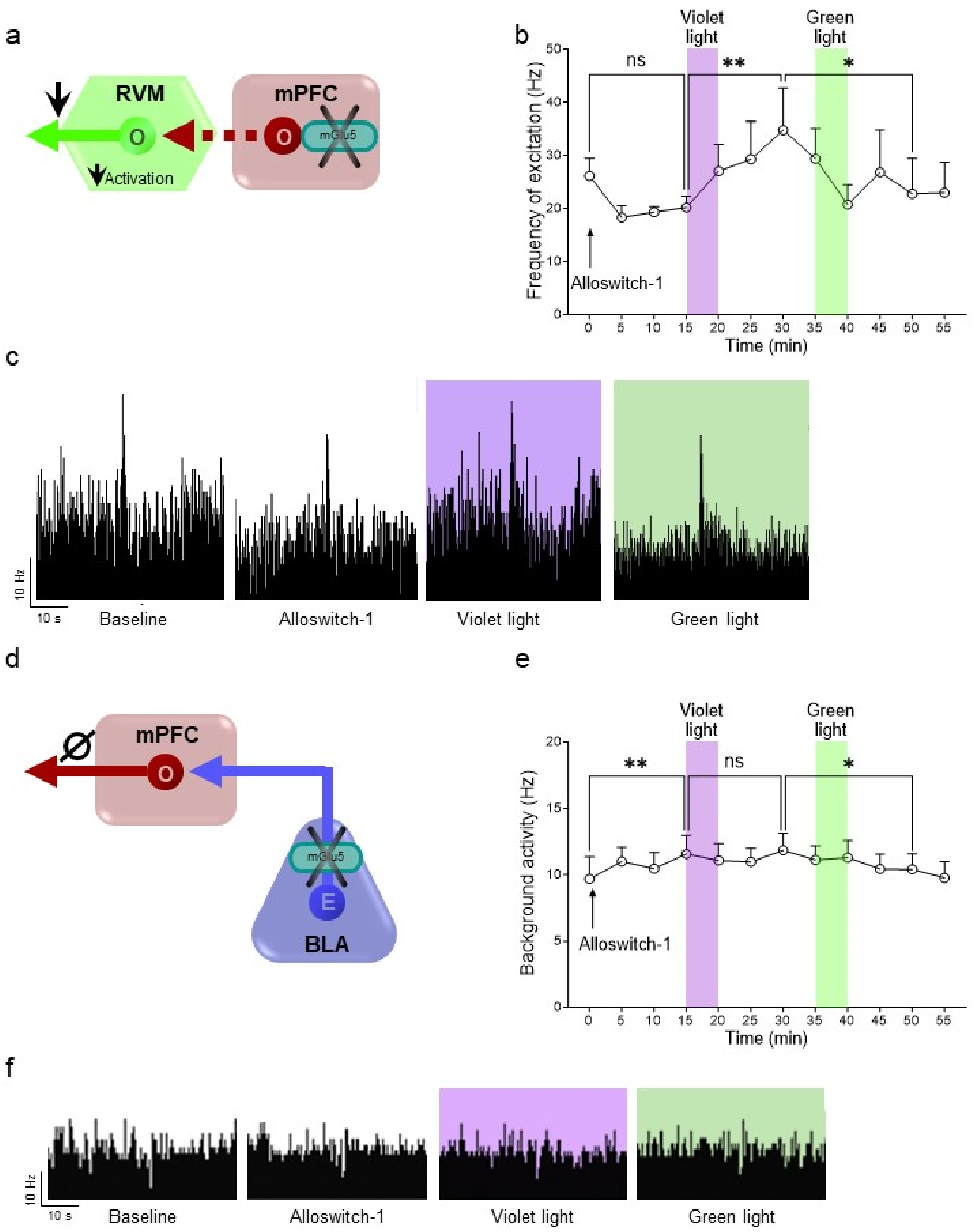

bioRxiv preprint doi: https://doi.org/10.1101/2024.01.02.573945; this version posted January 3, 2024. The copyright holder for this preprint (which was not certified by peer review) is the author/funder, who has granted bioRxiv a license to display the preprint in perpetuity. It is made available under aCC-BY 4.0 International license.

Fig. S3.

Effects of light-induced blockade of mGlu5 receptors in prelimbic cortex on descending pain control (RVM) and on BLA-prelimbic circuitry in CCI mice. (**a**) Single-unit recordings were performed from RVM ON-cells in anesthetized CCI mice 16 days after injury. An LED optic fiber was implanted into the contralateral (to the side of injury) prelimbic cortex for optical inactivation/activation of systemic alloswitch-1 (10 mg/kg, i.p.). (**b**) A trend of inhibition of RVM ON neuronal frequency of excitation was observed after alloswitch-1 systemic administration. Application of blue-violet light increased neuronal evoked activity while green light illumination restored burst of excitation in CCI anesthetized mice. (**c**) shows examples of ratemeter records that illustrate the light-dependent effects of alloswitch-1 on evoked neuronal activity of RVM ON neurons. To investigate the contribution of the BLA-prelimbic circuitry (**d**) to the facilitatory behavioral effects of mGlu5 inhibition in the amygdala (Fig. 2g, m), *in vivo* single unit recordings were performed from the prelimbic pyramidal neurons of CCI mice 16 days after injury. An LED optic fiber was implanted into the BLA contralateral (to the side of injury) to allow light-dependent deactivation/activation of alloswitch-1 (10 mg/kg, i.p.). Systemic administration of alloswitch-1 (10 mg/kg, i.p.) significantly increased background activity (**e**) of prelimbic neurons in neuropathic mice. Blue-violet light had no significant effects, while a decrease of activity was observed after green light illumination, which did not mimic the alloswitch-1 effects. (**f**) Shows examples of ratemeter records that illustrate the light-dependent effects of alloswitch-1 on spontaneous activity of prelimbic neurons. Each point represents the mean ± SEM of 4 (b) and 8 (e) neurons. One-way ANOVA repeated measures: (b) $F(11,44)=2.291$, $p=0.0257$; (e) $F(11,77)=2.724$, $p=0.005$. Bonferroni's multiple comparisons post hoc test. *$p<0.05$, **$p<0.01$.
